# Progress and Achievements in Glycosylation of Flavonoids

**DOI:** 10.3389/fchem.2021.637994

**Published:** 2021-03-31

**Authors:** Ruslana S. Khodzhaieva, Eugene S. Gladkov, Alexander Kyrychenko, Alexander D. Roshal

**Affiliations:** Institute of Chemistry, V. N. Karazin Kharkiv National University, Kharkiv, Ukraine

**Keywords:** glycosidic group, regioselectivity, chromone, glycosylation, flavonoid

## Abstract

In recent years, the chemistry of flavonoid glycosylation has undergone significant developments. This mini-review is devoted to summarizing existing strategies and methods for glycosylation of natural and synthetic flavonoids. Herein we overviewed the reaction conditions of flavonoid glycosylation depending on the position of hydroxyl groups in a parent molecule, the degree of it conjugation with the π-system, the presence of steric factors, the formation of intramolecular hydrogen bonds, etc. Especial attention was given to the choice of the glycosyl donor moiety, which has a significant effect on the yield of the final glycosidated products. Finally, a general strategy for regioselective glycosylation of flavonoids containing several hydroxyl groups is outlined.

## Introduction

Flavonoids are one of the most abundant of biological pigment classes, secondary metabolites that perform a wide variety of functions in plant and fungal organisms (Harborne and Baxter, 1999; Deveoglu and Karadag, 2019). The exact number of discovered and investigated flavonoids is currently unknown: various literature sources mention 500 (Havsteen, 1983) to 4,000 (Seigler, 1998; Harborne and Baxter, 1999) flavonoids of different structures and functions. This number is continuously growing so that some sources have recently reported more than 6,000 flavonoids (Stobiecki and Kachlicki, 2006). Common to all flavonoids is the presence of a chroman moiety, which has a substituted phenyl moiety at positions 2, 3, or 4, respectively. Depending on the position of the specified fragment, the presence or absence of a carbonyl group at the position 4 of the pyrane ring, a double bond between positions 2 and 3, as well as a positive charge, all flavonoids are divided into different classes (Havsteen, 1983; Harborne and Baxter, 1999; Panche et al., 2016). These classes may also include chalcones, which are precursors in the biosynthesis of the pigments, and aurones and coumestans that are often encountered as synthetic by-products (Hinderer and Seitz, 1988; Stobiecki and Kachlicki, 2006).

In plants, flavonoids are found in both aglycone and glycoside forms. However, the amount of glycosides significantly exceeds the number of glycoside-free forms (Hollman, 2004). Flavonol glycosides and aglycones perform various functions; in particular, they are responsible for UV photoprotection, reproduction, and internal regulation of plant cell physiology. Besides, they are scavengers of free radicals and contribute to the plant immune system (Falcone Ferreyra et al., 2012). No less diverse is the effect of flavonoids on other organisms. These compounds have the widest range of pharmacological effects and are natural pharmacological agents with significant therapeutic potential (Sridevi Sangeetha et al., 2016).

The usual way to study the structure or chemical analysis of flavonoids is their deglycosylation with subsequent determination of the structure or concentration of the aglycone component and the structure of the hydrocarbon moiety obtained as a result of hydrolysis (Pinheiro and Justino, 2012). However, this approach is hardly appropriate in the pharmaceutical chemistry of flavonoids. It is known that glycosides and aglycones exhibit different effects on organisms, and their therapeutic effect can differ significantly (Kumar and Pandey, 2013; Xiao, 2017; Alseekh et al., 2020). Moreover, currently there has been a need to solve the synthetic inverse problem - the creation of glycosides (at least O-glycosides) of new synthetic flavonoids and their heterocyclic derivatives with specific biological activity. Therefore, several studies are discussing methods for selective glycosylation of natural aglycones and the creation of new glycosides that are not encountered among natural analogues.

**GRAPHICAL ABSTRACT:**
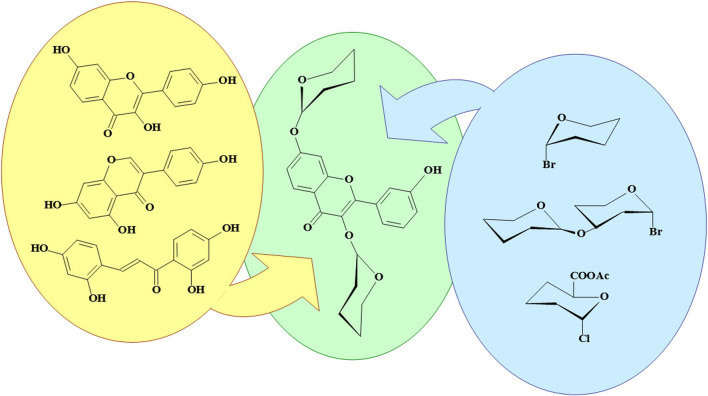


It is also known that flavonols and their heteroaryl-analogues are promising fluorescent probes (Demchenko et al., 2002; Roshal et al., 2003; Lvovskaya et al., 2006). Glycosylation of flavonols makes it possible to obtain a new class of fluorescent probes - glycosides with different spectral properties and increased solubility in polar media. Therefore, the targets of new probes in biological objects will differ from those of aglycon probes.

Summarizing the above arguments, we can conclude that flavonoid glycosylation is a promising direction for creating new drugs and fluorescent probes. This mini-review is devoted to the analysis of existing strategies and methods for glycosylation of natural and synthetic flavonoids.

Below we provide brief information on the strategy of glycosylation of flavonols, as well as on available donors of the glycosyl group and the most common reactions of glycosylation of flavonolides. A detailed description of the mechanisms of glycosylation reactions can be found elsewhere (Jensen, 2002; Das and Mukhopadhyay, 2016). Specific examples of glycosylation reactions of flavonoids are given in the following studies (Sun et al., 2014; Huanji et al., 2019).

## General Strategy for Glycosylation of Flavonoids

The large family of flavonoids includes compounds of entirely different chemical structures, with different reactivity and stability. This diversity leads to the fact that it is impossible to carry out a single standard glycosylation reaction for all compounds of the flavonoid family. Differences in the conditions of flavonoid glycosylation are mainly associated with the reactivity of hydroxyl groups due to their position in the molecule, the degree of conjugation with the π-system, the presence of steric factors, the formation of intramolecular hydrogen bonds, etc.

An essential factor that must be taken into account is the stability of flavonoids under the glycosylation reaction conditions. The process of direct glycosylation for some classes of flavonoids can lead to the destruction of the modified compounds. It should also be remembered that glycosylation is not limited to the attachment of the carbohydrate residue to the flavonoid part of the molecule, but is accompanied by the subsequent removal of the protective groups. This process can also lead to the partial destruction of some types of flavonoids (Needs and Williamson, 2001).

Considering the arguments mentioned above, it is clear that glycosylation of flavonoids cannot be a separate final stage in the production of glycosides. In some cases, it is closely intertwined with the synthesis of the flavonoid core itself.

At the first stage, various derivatives of 2-hydroxyacetophenone and benzaldehyde are condensed into the corresponding 2’-hydroxychalcone, which is the basic structure for further synthesis of flavonoids (Wagner and Farkas, 1975). Condensation can occur in both acidic and alkaline media, which in some cases makes it possible not to isolate chalcone, so that the flavonoid synthesis may be continued as a one-pot method in a wide pH range.

In the course of reactions in an acidic medium, the next stage is the cyclization of 2-hydroxychalcones into isomeric flavans, followed by oxidation into flavilium cations (Carbonneau et al., 2014). The anthocyanidins obtained in this way are extremely sensitive to the acidity of the medium and, when attempting to glycosylation, transform into an unstable tautomeric form capable of resinification. Obviously, in this case, glycosylation must be carried out at the stage of the initial products—benzaldehyde and acetophenone, or the intermediate product—2’-hydroxychalcone (Manna et al., 2019). This synthesis strategy is also applicable in the preparation of anthocyanidins from natural chalcones (Ngameni et al., 2008). In the synthesis of 5-hydroxyflavilium salts, acid condensation of phloroglucinol derivatives and benzoylacetone or benzoylpyruvic acid is used, bypassing the chalcone stage (Sweeny and Iacobucci, 1981). In this case, glycosylation is also desirable in the initial stages of the synthesis.

When carrying out the synthesis in an alkaline medium, chalcones in the presence of hydrogen peroxide are oxidized to α,β-epoxychalcones, which are subsequently recyclized into flavanonols, and then into flavonols (Algar–Flynn–Oyamada reaction) (Wang, 2010), as well as into aurones (Popova et al., 2019). Rearrangement of α,β-epoxychalcones into the corresponding formyldeoxybenzoins further leads to the formation of isoflavones (Szeja et al., 2017). Since reactions in an alkaline medium can lead to the hydrolysis of the acetyl protecting groups of the glycoside residue, the yield of the final glycoside will be minimal under such conditions. Therefore, when choosing the "alkaline" route for flavonoid synthesis, glycosylation should be carried out at the final stage of the synthesis, so that the obtained flavonoid is glycosidated directly.

As noted above, although some flavonoids successfully undergo direct glycosylation, the resulting glycosides can further decompose in alkaline media when the protective acetyl groups are removed. This reaction, for example, occurs in the case of isoflavones, which, upon alkaline deacylation, can partially decompose to deoxybenzoins (Needs and Williamson, 2001).

The earlier work found in the literature on the glycosylation of intermediates in the synthesis of flavonoids and the subsequent assembly of target compounds appears to be (Zemplén et al., 1943). The 4-(2”, 3”, 4”, 6”-tetra-O-acetyl)-O-β-D-glucopyranosyloxy flouracetophenone obtained in the first stage was further used to obtain glycosidated 2’-hydroxychalcone and, then, the corresponding flavanone.

**FIGURE 1:**
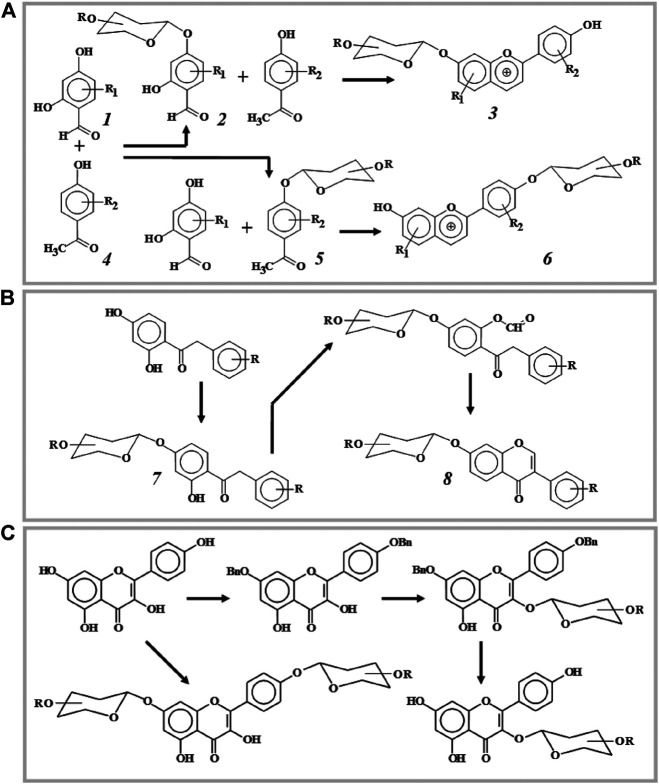
Three strategies of flavonoid glycosylation. **(A)**—glycosylation of starting reagents: anthocyanidine synthesis (regioselectivity of flavonoid synthesis is determined by glycosylation of one of the starting products). **(B)**—glycosylation of intermediate products: isoflavone synthesis (the regioselectivity of synthesis is determined by the direction of glycosylation of the intermediate). **(C)**—glycosylation of synthesis products: obtaining flavonol glucosides (the direction of glycosylation is regulated by the preliminary protection of certain hydroxyl groups of flavonol). 
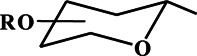
- β-D-glycosyl fragment with protected hydroxyl groups.

As an example of an "acidic" version of the synthesis, which provides for the glycosylation of the starting products and the subsequent assembly of flavonoid glycosides, one can mention the work (Al Bittar et al., 2016). The authors obtained various anthocyanidin glycosides based on 2,4-dihydroxybenzaldehyde (1) and 3,4-dihydroxy-acetophenone (4) (Figure 1A). Glycosylation of hydroxyl groups in both compounds is regioselective and occurs only with the hydroxyl group in the *para*-position of the benzene ring. The hydrogen-bound *ortho*-hydroxyl group has a lower acidity, and, therefore, for its glycosylation more tight conditions are required. Glycosylation of compound 1 and subsequent condensation of the obtained glycoside ***2*** with acetophenone ***4*** in the presence of trimethylchlorosilane leads to the formation of 7-(2", 3", 4", 6"-tetra-O-acetyl)-O-β-D-glucopyranosyloxy-4′-hydroxyflavylium chloride 3, i.e. anthocyanidin glycosylated in position 7. If acetophenone ***5*** is glycosylated and condensed with benzaldehyde 1, the resulting product is anthocyanidin 6, glycosylated at position 4′ of the side phenyl fragment. Similar reactions take place when there are different substituents in molecules 1 and 4, which makes it possible to obtain glycosides of various synthetic flavilium salts. This work demonstrates another advantage of the glycosylation strategy at the stage of initial reagents - glycosidic groups are introduced into the required positions of the molecule before its final assembly, which ensures a high regioselectivity of the reaction. An interesting result was obtained by the same authors in the synthesis of anthocyanidin based on phloroglucylic aldehyde glycoside (Mora-Soumille et al., 2013). Condensation of the latter with 3,4-dihydroxy acetophenone leads to the production of two products with an O-β-D-glycosyl group not only in 7, but also in 5 positions of the flavilium moiety.

Examples of glycosylation of chalcones, intermediates in the synthesis of many flavonoids, are given in (Ngameni et al., 2008). In this work, the authors described the glycylation and aminoglycylation of natural 4-hydroxychalcone—lonchocarpin and flavan in equilibrium with it.

Natural isoflavones ***8*** and their synthetic analogs can also be glycosylated at an intermediate stage of the "assembly" of the molecule (Figure 1B). For example, a glycoside moiety can be incorporated into the corresponding deoxybenzoin ***7*** (Pivovarenko et al., 1988a; Pivovarenko et al., 1989) or α-aryloxy-2,4-dihydroxyacetophenones (Pivovarenko et al., 1988b). In Wei and Yu (2008), isoflavone glucoside is obtained by glycosylation of the corresponding hydroxychromone followed by the introduction of a side benzene ring at position 3 of the chromone moiety.

At intermediate stages of flavonoid synthesis, it is possible to combine glycosylation with chemical modification of intermediate products, which makes it possible to obtain glycosides of a more complex chemical structure. For example, based on the cyclization reaction of azides with alkyne derivatives of chalcones, their triazole derivatives are obtained (Kant et al., 2016). The use of the azide group 2,3,4,6-tetra-O-acetyl-β-D-glucopyranosyl azide as a donor in the presence of CuSO_4_ and sodium ascorbate allows to obtain promising anti-cancer drugs—glucosides of triazole derivatives, in which the glycoside fragment is attached to heterocycle (Kant et al., 2016).

Most often, the glycosylation reaction is the last stage of the synthesis, when one or more glycosidic groups are condensed with the earlier-obtained aglycone (Figure 1C). As already noted, this strategy is used in the "alkaline" synthesis of flavonoids. Since the glycosylation of flavonoid aglycones is the most common reaction, it will be discussed in detail in the following sections.

## Donors of Glycosidic Groups

Although the nature of flavonoids plays an essential role in the choice of the conditions for glycosylation and the strategy for the synthesis of glycosides in general, the choice of the donor of the glycosyl group has a significant effect on the yield of the final products. The most successful and widespread is the use of the glucosyl residue 2,3,4,6-tetra-O-acetyl-β-D-glucopyranosyl bromide as a donor. The latter is synthesized by the method (Jeremias et al., 1948), and it is commercially available. Other halogen derivatives are less commonly used for glycosylation (Das and Mukhopadhyay, 2016; Arai et al., 2017). In works Li et al. (2008b), Yang et al. (2010), glucosyl *o*-hexynyl benzoates were used to obtain flavonoid glycosides in high yields.

In Hörhammer et al. (1966), the dependence of the yield of the glycosylation reaction of the flavonols, quercetin and isorhamnetin was investigated depending on the nature of the glycosyl residues. In particular, acetylated β-D-glycosyl bromides were used as monosaccharides with residues of glucose, galactose, xylose, rhamnose, and disaccharides - rutinose and cellobiose. It turned out that the yields of glycosides are in the range from ∼55% (for galactoside and rutinoside) up to ∼70–80% (for glucoside), while in the case of rhamnoside, the reaction yields do not exceed 1%. Similar studies, but using a different synthesis method, were carried out in (Demetzos et al., 1990). The yields for all glucosides ranged from 40–60%, with the exception of rhamnoside—not more than 10%. In addition, the formation of rhamnoside was accompanied by a loss of stereospecificity. Following the method proposed by (Hörhammer et al., 1966), the authors suggested that the problems with the synthesis of rhamnosides are due to the trans relationship between the activated Br and the participating CH_3_COO group in position 2. This is confirmed by the fact that the isomeric α-D-glucopyranosyl bromide is characterized by higher yields of the glycosylation reaction (Backinowsky et al., 1980). Studies of the influence of the type of glycosyl group on the glycosylation reactions of a series of synthetic flavonols are also described in (Li et al., 2008b). According to the data presented, glucosides and galactosides (57–77%) are formed with the highest yields, and xylosides and arabinosides (24–54%) with lower yields.

Also, in the synthesis of glycosides of flavonoids, oxidized forms are used, particularly bromides and chlorides of esters of glucuronic acid (Needs and Williamson, 2001; Reszka et al., 2020), as well as 2-acetamido-2-deoxy glucosides (Ngameni et al., 2008; Cao et al., 2018; Reszka et al., 2020). It should be noted that acetamides form final products with a higher yield than other donors of glycosidic groups—from 70–80% (Hörhammer et al., 1966) up to 92% (Reszka et al., 2020), respectively. The use of 2-acetamido-2-deoxy glucosides and glucuronides allows further chemical modification of glycosidic residues after removing the protective groups.

It is known that in the process of glycosylation reactions, glycoside residues undergo significant chemical transformations and form a large number of by-products (Christensen et al., 2015). Therefore, all hydroxyl groups of glycosides, the carboxyl group of the glucuronic residue, or the 2-amino group in amino sugars are previously protected. The classic method for protecting glycosidic residues in their acetylation. In addition, for the selective removal of protective groups at the end of glycosylation of flavonoids, some hydroxyl groups in glycosides are benzylated (Maloney and Hecht, 2005) or benzoylated (Li et al., 2008a; Yang et al., 2010).

Despite the presence of protective groups, some of the glycosyl bromides undergo hydrolysis, which is why the glycosylation reaction in some cases must be carried out in additionally dehydrated solvents and the presence of a water adsorbent, for example, molecular sieves (Needs and Williamson, 2001; Smith et al., 2006; Mei et al., 2015). Moreover, some authors note the appearance in the reaction mixture of side products—glycals (Needs and Williamson, 2001; Reszka et al., 2020).

## Flavonoid Glycosylation Reactions

For the glycosylation of flavonoids, a variety of four main methods are used, some of which were developed from the late 30s to the early 50s of the last century.

**FIGURE 2:**
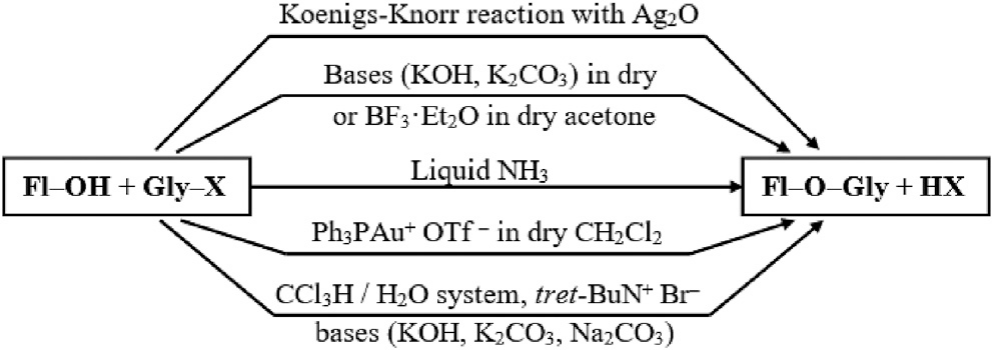
Main pathways of flavonoid synthesis.

The first method, most likely, should be considered the one described in the work of S.-I. Fujitse and S. Mitsui (Fujise and Mitui, 1938) the Koenigs–Knorr reaction (Koenigs and Knorr, 1901) adapted to flavonoids, in which, instead of the acceptor of bromine atoms, Ag_2_CO_3_, activated Ag_2_O was used (Figure 2). The reaction between the flavanone derivatives and 2,3,4,6-tetra-O-acetyl-a-D-glucopyranosyl bromide was carried out in a mixture of quinoline and benzene. In later works, glycosylation was carried out in pure quinoline (Zemplén et al., 1943), pyridine (Hörhammer et al., 1966), chloroform with the addition of a desiccant (Maloney and Hecht, 2005; Smith et al., 2006), and freshly distilled dichloromethane (Mei et al., 2015). The reaction has been used to glycosylate flavanones, chalcones, and flavonols, respectively. The reaction yield usually ranges from 30% up to 70%, depending on the nature of the glycosylated flavonoid.

Attempts to obtain flavonol glycosides using the classical Köning-Knorr reaction in the presence of Ag_2_CO_3_ failed (Needs and Williamson, 2001; Mei et al., 2015). The glycosylation of isoflavones in dichloromethane in the presence of Ag_2_CO_3_, collidine, and a desiccant made it possible to obtain the final product with a yield of 10% in the presence of significant amounts of a by-product, glycal (Needs and Williamson, 2001; Cao et al., 2018). Attempts to use SrCO_3_ or AgNO_3_ salts in the presence of pyridine and quinoline as acceptors of bromine ions also turned out to be unsuccessful or unreproducible (Li et al., 2006; Wang and Zhang, 2011).

Another method, which has been used in flavonoid chemistry since the 1940s, is the conventional esterification reaction in an anhydrous solvent in the presence of a base or a Lewis acid. The reaction is carried out in dry acetone; KOH (Hörhammer et al., 1966) or K_2_CO_3_ (Neves et al., 2018) is used as alkali. The yield of flavonol glycosides is low and amounts to 5–10%. In the case of glycosylation of 5,7,4’-trihydroxyisoflavone in the presence of NaOH, the glycoside was obtained with a yield of ∼65% (Zemplén and Farkas, 1943). The use of Lewis acid BF_3_·Et_2_O for the esterification of the catalyst in dichloromethane at −15°C in an argon atmosphere made it possible to carry out the reaction of 7,4’-dihydroxyisoflavone and methyl 2,3,4-tri-O-acetyl-α-D-glucopyranosyluronate bromide with a yield of 14% (Needs and Williamson, 2001).

Attempts to glycosylate 3,5,7,3′, 4′-pentahydroxy flavone (quercetin) with hexa-O-acetyl-α-D-glucorutinosyl bromide (Samokhvalov et al., 1958) and tetra-O-acetyl-α-D-glucopyranosyl bromide (Ice and Wender, 1952) were made in liquid ammonia. The reaction was carried out with the sodium salt of quercetin. The reaction components were dissolved in liquid ammonia, the spontaneous evaporation of ammonia was waited for, and then the resulting residue was kept in methanol for a day. The glycoside yield was about 30%.

The most popular method is the synthesis of glycosides under phase-transfer conditions in the chloroform-water system. Apparently, this method was first tested for flavonoids in (Demetzos et al., 1990). The donor of the glycoside group, polyhydroxyflavonol, and a phase transfer catalyst—benzyltriethylammonium bromide, were stirred in the organic phase, and then a dilute aqueous KOH solution was added to it. The two-phase system chloroform water 5: 2 v/v was intensively stirred and boiled for 15 h. The yields of flavonol glycosides were 40–60%. The same method was used for the glycosylation of simple phenols (Pavlov et al., 2001). In this case, the yield of phenols was from 12% up to 44%, respectively.

To increase the yield of the final product, instead of KOH, NaOH (Zhu et al., 2006) and also less alkaline agents - Na_2_CO_3_ (Mughal et al., 2018) or K_2_CO_3_ (Semeniuchenko et al., 2009; Serdiuk et al., 2016; Reszka et al., 2020) began to be used. The yield of flavonols-glycosides with donors of glycoside groups, uronates, and 2-acetamides was 40–95%.

In contrast to flavonols, the yields of glycosides of chalcones remained relatively low about 20–30%. An increase in the yield of chalcone glycosides up to 43–61% was achieved using ethyl acetate as a non-aqueous phase, an alkaline agent—Na_2_CO_3_, and a catalyst—*tert*-butylammonium hydrogen sulfate (Ngameni et al., 2008).

The relatively recent method of glycosylation of flavonols is interaction with glycosyl *o*-hexynylbenzoates using a monovalent gold salt as a catalyst, namely Ph_3_PAuOTf (triphenylphosphinegold (I) bis(trifluoromethane) sulfonimide) in dichloromethane in the presence of molecular sieves as a desiccant (Li et al., 2008a; Yang et al., 2010). The glycoside yield was 80–95%. For the reaction to take place, it is necessary to protect all hydroxyl groups of flavonols, including the low-activity 5-hydroxy group. An important condition for successful glycosylation is the presence of a protective benzoyl group at position 2 of the pyranose ring. When using the protective acetyl group, glycosylation does not occur.

The last stage in the synthesis of flavonoid glycosides is the hydrolysis of the protective groups of the glycosidic residue. When obtaining flavanones, flavones, and flavonols, removal of protective acetyl groups with sodium methanolate or NaOH in methanol or chloroform-methanol media at room or low temperatures (Fujise and Mitui, 1938; Hörhammer et al., 1966). The acetyl protection can also be removed by dissolving the glycoside in a 7M methanol solution of ammonia (Mei et al., 2015). In this case, complete separation of protective groups occurs in ∼3 h at room temperature.

Such hydrolysis methods are not suitable for isoflavones: treatment with even such a weak base as LiOH leads to partial decomposition of the γ-pyrone ring and the formation of deoxybenzoins. In this case deacylation is carried out in a water-methanol solution of Na_2_CO_3_ at room temperature (Needs and Williamson, 2001).

## Regioselectivity of Glycosylation Reactions

Natural flavonoids contain, as a rule, from two to five hydroxyl groups, so the question of the direction of glycosylation reactions is of great importance. As noted earlier, the easiest way to ensure the required position of the glycosidic group is to obtain the glycosides of the starting reagents.

The tendency to form glycosides is determined by the acidity of hydroxyl groups, which can vary from 7.5–12 pH units (Escandar and Sala, 1991; Musialik et al., 2009). By acidity, and, accordingly, by activity in glycosylation reactions, hydroxyl groups of flavones and isoflavones are distributed as follows: 7-OH ≥ 4’-OH> 3-OH > 3′-OH> 5-OH. Since most acidic are 7- and 4’-hydroxyl groups, therefore, to obtain 7-O-glycosides, hydroxyl group 4’ should be protected. It is known, however, that in the case of 7,4’′-dihydroxy isoflavone in the presence of Ag_2_O, only the 7-hydroxyl group is glycosylated, while when BF_3_ is used, a diglycoside is formed (Needs and Williamson, 2001). Polyhydroxyflavones, such as 5,7,4’-trihydroxyflavone, form diglycosides at 7- and 4’-hydroxyl groups (Demetzos et al., 1990), while 3,7-dihydroxyflavone results in 3,7-O-diglycoside accompanied with a small amount of 7-O-glycoside (Neves et al., 2018).

In the synthesis of 3-O-glycosides of polyhydroxyflavone, such as rutin, all other hydroxyl groups except 5-OH are benzylated (Cao et al., 2018). The 5-OH group exhibits the lowest acidity due to the formation of an intramolecular hydrogen bond with the carbonyl group and is usually not glycosylated. Therefore, in the synthesis of glycosides, this group is not protected. Pathways of the selective glycosylation of flavonol kaempferol are presented in Figure 1C.

There are no special works on the analysis of the regioselectivity of glycosylation reactions of flavonoids; however, due to the presence of the need for obtaining glycosides of a given structure, this issue nowadays remains relevant and requires additional research.

## Summary and Perspectives

In this mini-review, we summarized existing glycosylation strategies and synthetic methods for glycosylation of natural and synthetic flavonoids. In our opinion, if doubts usually do not arise with the definition of the synthesis strategy, then it is not possible to single out any one best method of glycosylation. The choice of the glycosylation method and the donor of the glycoside group is due to the presence of previous practical experience, and attempts to modify the reaction conditions can often be explained by the manifestation of the “chemical intuition”.

Among the methods considered, it seems that glycosylation by direct esterification in dry acetone and liquid ammonia can be discarded, since these methods give glycosides in low yields. The method using monovalent gold as a catalyst requires preliminary synthesis of glycosyl hexynylbenzoates and the protection of 2-hydroxyl groups of the glycoside with a benzoate group. In addition, complete protection of flavonoid hydroxyl groups and complete dehydration of the reaction medium are required. Perhaps this rather laborious method can be used in any special cases.

The most convenient are glycosylation using phase-transfer in the chloroform-water system, as well as the method based on the Koenigs–Knorr reaction. Both methods give approximately the same yields of the target products. However, it should be noted that the latter method seems to be less preferable due to the need to use large amounts of silver oxide and the need for its further disposal. Moreover, this method is extremely sensitive to the presence of moisture and requires deep drying of the reaction medium. Therefore, for the glycosylation of flavonoids, the authors of this mini-review have chosen and use the phase-transfer method.

## References

[B1] Al Bittar S. Mora N. Loonis M. Dangles O. (2016). A simple synthesis of 3-deoxyanthocyanidins and their O-glucosides. Tetrahedron 72 (29), 4294–4302. 10.1016/j.tet.2016.05.076

[B2] Alseekh S. Perez de Souza L. Benina M. Fernie A. R. (2020). The style and substance of plant flavonoid decoration; towards defining both structure and function. Phytochemistry 174, 112347. 10.1016/j.phytochem.2020.112347 32203741

[B3] Arai M. A. Yamaguchi Y. Ishibashi M. (2017). Total synthesis of agalloside, isolated from *Aquilaria agallocha*, by the 5-O-glycosylation of flavan. Org. Biomol. Chem. 15 (23), 5025–5032. 10.1039/C7OB01004D 28569322

[B4] Backinowsky L. V. Balan N. F. Shashkov A. S. Kochetkov N. K. (1980). Synthesis and 13C-NMR spectra of β-l-rhamnopyranosides. Carbohydr. Res. 84 (2), 225–235. 10.1016/S0008-6215(00)85553-6

[B5] Cao Z. Chen J. Zhu D. Yang Z. Teng W. Liu G. (2018). Regiospecific synthesis of three quercetin O-β-Glucosides of N-Acetylglucosamine. J. Chem. Res. 42 (4), 189–193. 10.3184/174751918X15232706115112

[B6] Carbonneau M.-A. Cisse M. Mora-Soumille N. Dairi S. Rosa M. Michel F. (2014). Antioxidant properties of 3-deoxyanthocyanidins and polyphenolic extracts from Côte d’Ivoire’s red and white sorghums assessed by ORAC and *in vitro* LDL oxidisability tests. Food Chem. 145, 701–709. 10.1016/j.foodchem.2013.07.025 24128534

[B7] Christensen H. M. Oscarson S. Jensen H. H. (2015). Common side reactions of the glycosyl donor in chemical glycosylation. Carbohydr. Res. 408, 51–95. 10.1016/j.carres.2015.02.007 25862946

[B8] Das R. Mukhopadhyay B. (2016). Chemical O-glycosylations: An overview. Chem. Open 5 (5), 401–433. 10.1002/open.201600043 PMC5062006 27777833

[B9] Demchenko A. P. Ercelen S. Roshal A. D. Klymchenko A. S. (2002). Excited-state proton transfer reaction in a new benzofuryl 3-hydroxychromone derivative: The influence of low-polar solvents. Polish J. Chem. 76 (9), 1287–1299.

[B10] Demetzos C. Skaltsounis A.-L. Tillequin F. Koch M. (1990). Phase-transfer-catalyzed synthesis of flavonoid glycosides. Carbohydr. Res. 207 (1), 131–137. 10.1016/0008-6215(90)80012-R 2076512

[B11] Deveoglu O. Karadag R. (2019). A Review on the flavonoids – a dye source. Int. J. Adv. Eng. Pure Sci. 3, 188–200. 10.7240/jeps.476514

[B12] Escandar G. M. Sala L. F. (1991). Complexing behavior of rutin and quercetin. Can. J. Chem. 69 (12), 1994–2001. 10.1139/v91-288

[B13] Falcone Ferreyra M. L. Rius S. Casati P. (2012). Flavonoids: Biosynthesis, biological functions, and biotechnological applications. Front. Plant Sci. 3, 222. 10.3389/fpls.2012.00222 23060891 PMC3460232

[B14] Fujise S.-I. Mitui S. (1938). Versuche zur synthese von oxyflavanon-glucosiden. Ber. Dtsch. Chem. Ges. A/B 71 (4), 912–915. 10.1002/cber.19380710434

[B15] Harborne J. B. Baxter H. (1999). The handbook of natural flavonoids. Chichester: John Wiley and Sons.

[B16] Havsteen B. (1983). Flavonoids, a class of natural products of high pharmacological potency. Biochem. Pharmacol. 32 (7), 1141–1148. 10.1016/0006-2952(83)90262-9 6342623

[B17] Hinderer W. Seitz H. U. (1988). “Flavonoids,” in Phytochemicals in plant cell cultures. Editors Constabel F., and Vasil I. K. (New York, NY: Academic Press), 23–48.

[B18] Hollman P. C. H. (2004). Absorption, bioavailability, and metabolism of flavonoids. Pharm. Biol. 42 (1), 74–83. 10.3109/13880200490893492

[B19] Hörhammer L. Wagner H. Arndt H. G. Kraemer H. Farkas L. (1966). Syhthese natürlich vorkommender polyhydroxy-flavonol-glykoside. Tetrahedron Lett. 7 (6), 567–571. 10.1016/s0040-4039(01)99666-2

[B20] Huanji X. Zheming L. Yunqiu W. Di L. Li Q. Jizhao X. (2019). Advances on synthesis of flavonoid glycosides. Chin. J. Org. Chem. 39 (7), 1875–1890. 10.6023/cjoc201811002

[B21] Ice C. H. Wender S. H. (1952). The Synthesis of isoquercitrin. J. Am. Chem. Soc. 74 (18), 4606. 10.1021/ja01138a048

[B22] Jensen K. J. (2002). O-Glycosylations under neutral or basic conditions. J. Chem. Soc. Perkin Trans. 1 (20), 2219–2233. 10.1039/B110071H

[B23] Jeremias C. G. Lucas G. B. MacKenzie C. A. (1948). Preparation of tetraacetyl-α-D-glucopyranosyl bromide. J. Am. Chem. Soc. 70 (7), 2598. 10.1021/ja01187a507 18875126

[B24] Kant R. Kumar D. Agarwal D. Gupta R. D. Tilak R. Awasthi S. K. (2016). Synthesis of newer 1,2,3-triazole linked chalcone and flavone hybrid compounds and evaluation of their antimicrobial and cytotoxic activities. Eur. J. Med. Chem. 113, 34–49. 10.1016/j.ejmech.2016.02.041 26922227

[B25] Koenigs W. Knorr E. (1901). Ueber einige derivate des traubenzuckers und der galactose. Ber. Dtsch. Chem. Ges. 34 (1), 957–981. 10.1002/cber.190103401162

[B26] Kumar S. Pandey A. K. (2013). Chemistry and biological activities of flavonoids: An overview. Sci. World J. 2013, 162750. 10.1155/2013/162750 PMC3891543 24470791

[B27] Li Y.-L. Zhu Y.-C. Cui L.-Y. (2006). Total synthesis method of natural product barrenwort glycosides compounds. China patent application CN20061165354.

[B28] Li Y. Yang Y. Yu B. (2008a). An efficient glycosylation protocol with glycosyl ortho-alkynylbenzoates as donors under the catalysis of Ph_3_PAuOTf. Tetrahedron Lett. 49 (22), 3604–3608. 10.1016/j.tetlet.2008.04.017

[B29] Li Z. Ngojeh G. DeWitt P. Zheng Z. Chen M. Lainhart B. (2008b). Synthesis of a library of glycosylated flavonols. Tetrahedron Lett. 49 (51), 7243–7245. 10.1016/j.tetlet.2008.10.032 32287439 PMC7111846

[B30] Lvovskaya M. I. Roshal A. D. Doroshenko A. O. Kyrychenko A. V. Khilya V. P. (2006). Fluorescence behavior of chromones containing several protolytic centers. 3-Thiazolylchromones: Emission band assignment and pH dependent effects. Spectrochim. Acta A Mol. Biomol. Spectrosc. 65 (2), 397–405. 10.1016/j.saa.2005.11.020 16533617

[B31] Maloney D. J. Hecht S. M. (2005). Synthesis of a potent and selective inhibitor of p90 Rsk. Org. Lett. 7 (6), 1097–1099. 10.1021/ol0500463 15760148

[B32] Manna T. Pal K. Jana K. Misra A. K. (2019). Anti-cancer potential of novel glycosylated 1,4-substituted triazolylchalcone derivatives. Bioorg. Med. Chem. Lett. 29 (19), 126615. 10.1016/j.bmcl.2019.08.019 31447083

[B33] Mei Q. Wang C. Zhao Z. Yuan W. Zhang G. (2015). Synthesis of icariin from kaempferol through regioselective methylation and para-Claisen–Cope rearrangement. Beilstein J. Org. Chem. 11, 1220–1225. 10.3762/bjoc.11.135 26425179 PMC4578360

[B34] Mora-Soumille N. Al Bittar S. Rosa M. Dangles O. (2013). Analogs of anthocyanins with a 3’,4’-dihydroxy substitution: Synthesis and investigation of their acid–base, hydration, metal binding and hydrogen-donating properties in aqueous solution. Dyes Pigm. 96 (1), 7–15. 10.1016/j.dyepig.2012.07.006

[B35] Mughal E. U. Javid A. Sadiq A. Murtaza S. Zafar M. N. Khan B. A. (2018). Synthesis, structure-activity relationship and molecular docking studies of 3-O-flavonol glycosides as cholinesterase inhibitors. Bioorg. Med. Chem. 26 (12), 3696–3706. 10.1016/j.bmc.2018.05.050 29886083

[B36] Musialik M. Kuzmicz R. Pawłowski T. S. Litwinienko G. (2009). Acidity of hydroxyl groups: An overlooked influence on antiradical properties of flavonoids. J. Org. Chem. 74 (7), 2699–2709. 10.1021/jo802716v 19275193

[B37] Needs P. W. Williamson G. (2001). Syntheses of daidzein-7-yl β-d-glucopyranosiduronic acid and daidzein-4′,7-yl di-β-d-glucopyranosiduronic acid. Carbohydr. Res. 330 (4), 511–515. 10.1016/S0008-6215(00)00326-8 11269403

[B38] Neves A. R. Correia-da-Silva M. Silva P. M. A. Ribeiro D. Sousa E. Bousbaa H. (2018). Synthesis of new glycosylated flavonoids with inhibitory activity on cell growth. Molecules 23 (5), 1093. 10.3390/molecules23051093 PMC6102538 29734739

[B39] Ngameni B. Patnam R. Sonna P. Ngadjui B. T. Roy R. Abegaz B. M. (2008). Hemisynthesis and spectroscopic characterization of three glycosylated 4-hydroxylonchocarpins from dorstenia barteri bureau. ARKIVOC 2008 (6), 152–159. 10.3998/ark.5550190.0009.614

[B40] Panche A. N. Diwan A. D. Chandra S. R. (2016). Flavonoids: An overview. J. Nutr. Sci. 5, e47. 10.1017/jns.2016.41 28620474 PMC5465813

[B41] Pavlov A. E. Sokolov V. M. Zakharov V. I. (2001). Structure and reactivity of glycosides: IV. Koenigs-Knorr synthesis of aryl β-D-glucopyranosides using phase-transfer catalysts. Russ. J. Gen. Chem. 71 (11), 1811–1814. 10.1023/A:1013971214679

[B42] Pinheiro P. F. Justino G. C. (2012). “Structural analysis of flavonoids and related compounds - a review of spectroscopic applications,” in Phytochemicals - a global perspective of their role in nutrition and health. Editor Rao V. (New York,NY: IntechOpen), 33–56.

[B43] Pivovarenko V. G. Khilya V. P. Kovalev V. N. Vasil'ev S. A. (1988a). Effective synthesis of 7-hydroxyisoflavone O-glucosides. Chem. Nat. Compd. 24 (4), 432–438. 10.1007/BF00598526

[B44] Pivovarenko V. G. Khilya V. P. Kovalev V. N. Vasil'ev S. A. (1988b). Synthesis of α-aryloxy-2,4-dihydroxyacetophenone 4-O-β-D-glucopyranosides and their conversion into 3-aryloxy-7-glucosyloxychromones. Chem. Nat. Compd. 24 (4), 439–443. 10.1007/BF00598527

[B45] Pivovarenko V. G. Khilya V. P. Vasil’ev S. A. (1989). Simple and effective synthesis of isoflavones and 3-aryloxychromones. Chem. Nat. Compd. 25 (5), 542–545. 10.1007/BF00598071

[B46] Popova A. V. Bondarenko S. P. Frasinyuk M. S. (2019). Aurones: Synthesis and properties. Chem. Heterocycl. Comp. 55 (4), 285–299. 10.1007/s10593-019-02457-x

[B47] Reszka M. Serdiuk I. E. Kozakiewicz K. Nowacki A. Myszka H. Bojarski P. (2020). Influence of a 4’-substituent on the efficiency of flavonol-based fluorescent indicators of β-glycosidase activity. Org. Biomol. Chem. 18 (38), 7635–7648. 10.1039/D0OB01505A 32960207

[B48] Roshal A. D. Organero J. A. Douhal A. (2003). Tuning the mechanism of proton-transfer in a hydroxyflavone derivative. Chem. Phys. Lett. 379 (1), 53–59. 10.1016/j.cplett.2003.08.008

[B49] Samokhvalov G. I. Shakhova M. K. Preobrazhenski N. A. (1958). The synthesis of rutin. Dokl. Acad. Nauk SSSR 123, 305–307.

[B50] Seigler D. S. (1998). “Flavonoids,” in Plant secondary metabolism. Editor Seigler D. S. (Boston, MA: Springer), p. 151–192.

[B51] Semeniuchenko V. Garazd Y. Garazd M. Shokol T. Groth U. Khilya V. (2009). Highly efficient glucosylation of flavonoids. Monatsh. Chem. 140 (12), 1503. 10.1007/s00706-009-0207-6

[B52] Serdiuk I. E. Reszka M. Myszka H. Krzymiński K. Liberek B. Roshal A. D. (2016). Flavonol-based fluorescent indicator for determination of β-glucosidase activity. RSC Adv. 6 (48), 42532–42536. 10.1039/C6RA06062E

[B53] Smith J. A. Maloney D. J. Clark D. E. Xu Y. Hecht S. M. Lannigan D. A. (2006). Influence of rhamnose substituents on the potency of SL0101, an inhibitor of the Ser/Thr kinase, RSK. Bioorg. Med. Chem. 14 (17), 6034–6042. 10.1016/j.bmc.2006.05.009 16723233

[B54] Sridevi Sangeetha K. S. Umamaheswari S. Uma Maheswara Reddy C. Narayana Kalkura S. (2016). Flavonoids: Therapeutic potential of natural pharmacological agents. Int. J. Pharm. Sci. Res. 7 (10), 3924–3930. 10.13040/IJPSR.0975-8232.7(10).3924-3010.22376/ijpbs.2016.7.4.b106-114

[B55] Stobiecki M. Kachlicki P. (2006). “Isolation and identification of flavonoids,” in The science of flavonoids. Editor Grotewold E. (New York, NY: Springer), p. 47–69.

[B56] Sun J. Laval S. Yu B. (2014). Glycosylation reactions in the synthesis of flavonoid glycosides. Synthesis 46 (08), 1030–1045. 10.1055/s-0033-1341052

[B57] Sweeny J. G. Iacobucci G. A. (1981). Synthesis of anthocyanidins-III: Total synthesis of apigeninidin and luteolinidin chlorides. Tetrahedron 37 (8), 1481–1483. 10.1016/S0040-4020(01)92086-1

[B58] Szeja W. Grynkiewicz G. Rusin A. (2017). Isoflavones, their glycosides and glycoconjugates. synthesis and biological activity. Curr. Org. Chem. 21 (3), 218–235. 10.2174/1385272820666160928120822 28553156 PMC5427819

[B59] Wagner H. Farkas L. (1975). “Synthesis of flavonoids,” in The flavonoids. Editors Harborne J. B. Mabry T. J., and Mabry H. (Boston, MA: Springer), p. 127–213.

[B60] Wang B.-B. Zhang S. (2011). Method for synthesizing icariin by glucosidation of dehydrated epimedium herb. China patent application CN102093450.

[B61] Wang Z. (2010). “Algar-flynn-oyamada (AFO) reaction,” in Comprehensive organic name reactions and reagents. Editor Wang Z. (London: John Wiley and Sons, Inc), 52–56.

[B62] Wei G. Yu B. (2008). Isoflavone glycosides: Synthesis and evaluation as α-glucosidase inhibitors. Eur. J. Org. Chem. 2008 (18), 3156–3163. 10.1002/ejoc.200800239

[B63] Xiao J. (2017). Dietary flavonoid aglycones and their glycosides: Which show better biological significance?. Crit. Rev. Food Sci. Nutr. 57 (9), 1874–1905. 10.1080/10408398.2015.1032400 26176651

[B64] Yang W. Sun J. Lu W. Li Y. Shan L. Han W. (2010). Synthesis of Kaempferol 3-O-(3”,6”-Di-O-E-p-coumaroyl)-β-d-glucopyranoside, efficient glycosylation of flavonol 3-OH with glycosyl o-alkynylbenzoates as donors. J. Org. Chem. 75 (20), 6879–6888. 10.1021/jo1014189 20839821

[B65] Zemplén G. Bognár R. Szegö L. (1943). Synthese des Eriodictyols und des 3-Oxy-p-phlorrhizins. Ber. Dtsch. Chem. Ges. A/B 76 (11), 1112–1115. 10.1002/cber.19430761106

[B66] Zemplén G. Farkas L. (1943). Synthese des genistins. Ber. Dtsch. Chem. Ges. A/B 76 (11), 1110–1112. 10.1002/cber.19430761105

[B67] Zhu C. Peng W. Li Y. Han X. Yu B. (2006). Synthesis of 3-O-(β-d-xylopyranosyl-(1→2)-β-d-glucopyranosyl)-3’-O-(β-d-glucopyranosyl)tamarixetin, the putative structure of aescuflavoside A from the seeds of *Aesculus chinensis* . Carbohydr. Res. 341 (8), 1047–1051. 10.1016/j.carres.2006.02.036 16580652

